# Integrating batch marks and radio tags to estimate the size of a closed population with a movement model

**DOI:** 10.1002/ece3.876

**Published:** 2013-11-14

**Authors:** Carl James Schwarz, Scott Cope, Glenda Fratton

**Affiliations:** 1 Statistics and Actuarial Science, Simon Fraser UniversityBurnaby, BC, V5A 1S6, Canada; 2 Westslope Fisheries Ltd.800 Summit Drive, Cranbrook, BC, V1C 5J5, Canada; 3 Teck Coal LimitedSuite 1000, 205 9th Avenue SE, Calgary, AB, T2G 0R3, Canada

**Keywords:** Batch-marking, Capture–recapture, movement.

## Abstract

Movement models require individually identifiable marks to estimate the movement rates among strata. But they are relatively expensive to apply and monitor. Batch marks can be readily applied, but individual animal movements cannot be identified. We describe a method to estimate population size in a stratified population when movement takes place among strata and animals are marked with a combination of batch and individually identifiable tags. A hierarchical model with Bayesian inference is developed that pools information across segments on the detection efficiency based on radio-tagged fish and also uses the movement of the radio-tagged fish to impute the movement of the batch-marked fish to provide estimates of the population size on a segment and river level. The batch marks provide important information to help estimate the movement rates, but contribute little to the overall estimate of the population size. In this case, the approximate equal catchability among strata in either sample obviates the need for stratification.

## Introduction

Two-sample mark–recapture experiments are commonly used to estimate the population size of closed populations. A key assumption of the Lincoln-Petersen estimator commonly used for these experiments is that the probability of capture at each sampling event is homogeneous across the population, and that all tags are available for recapture. Failure of these assumptions can lead to the estimates of population size that are biased.

Schaefer (1951), Chapman and Junge (1956), and Darroch (1961) were among the first to deal with heterogeneity in catchability through the stratification of releases and recaptures into homogeneous strata when animals are not constrained to remain in the stratum of release. Stratification can be temporal or geographical. Plante et al. (1998) extended the above work to deal with cases when the number of release and recovery strata differed and Arnason et al. (1996) provided easy-to-use software.

Arnason (1972, 1973) examined the case of more than 2 sample times; Schwarz and Ganter (1995) provided a general formulation for closed populations; and Dupuis and Schwarz (2007) extended this to open populations.

More recently, Bayesian methods have been introduced for the two sample problem to share data among strata with sparse counts (Mäntyniemi and Romakkaniemi 2002) and to impose a regular structure on the movements so that strata with the missing data can be accommodated (Bonner and Schwarz 2011).

All of the above methods assume that animals are marked with tags to identify individuals so that the stratum of release and recapture can be identified. This does not usually cause any restrictions in the sampling protocol if animals are easily handled, tags are easy to apply and to read, or if the tag number can be read at a distance (e.g., radio tags). However, in experiments with fish, tagging may be difficult and batch marks (e.g., fin clips) used instead. Also, in some studies, fish are observed at a distance (e.g., snorkel surveys) and the individual tag numbers may be hard to read.

Batch marks can be used to separate marked from unmarked fish, but provide no information on the movement of individual fish. Huggins et al. (2010) and Cowen et al. (2013) considered a problem where fish were batch-marked at each sampling occasion so that the capture histories of individual fish could not be obtained. Huggins et al. (2010) used a marginal likelihood approach while Cowen et al. (2013) computed an exact likelihood to estimate survival and catchability. Both used a Horwitz–Thompson-like estimator for abundance.

In this paper, we consider the case of estimating the size of a closed population of fish in a two-sample stratified (segments of a river) experiment where fish can move among the strata between the sampling events. Two types of tags were used in the experiment. First, radio tags were applied to some fish, but this is an expensive procedure because surgery must be performed on the fish to implant the radio tags. However, the radio tags can be read from a distance (the shore). Second, Floy tags were also applied to the remainder (the majority) of the captured fish. Because the river is relatively small in our application, snorkel surveys were used on the second sampling occasion to search for fish. Detection is less than 100%, and the capture efficiency of the snorkel team was estimated by the fraction of the (known numbers of) radio-tagged fish that were detected in the segment of the river. We adopt a hierarchical framework with Bayesian inference because of the sparseness of the data and to share information among strata.

## Methods

### Sampling protocol

The Fording River in southeast British Columbia contains one of the few remaining streams with a genetically pure strain of Westslope Cutthroat Trout (*Oncorhynchus clarkii lewisi*). A waterfall provides a natural barrier to upward migration; therefore, the Westslope cutthroat trout population of concern is considered a resident, fluvial, headwater population restricted to the approximately 60 km of the river above the falls.

The full sampling protocol is explained in Cope et al. (2013). Briefly, the river was divided into 12 segments, each approximately 5 km long, based on the natural features of the river. In early August and September 2012, just over 230 adult and subadult fish were captured. About one-fourth (60 fish) were implanted with radio tags and a unique (green) numbered Floy tag. The remaining 172 fish were simply tagged with a unique (white) Floy tag. Both types of tagged fish were allowed to recover and then released back into the stream near where they were captured.

About 3 weeks later, snorkel surveys were carried out one reach at a time and surveyors recorded the numbers of fish seen with green tags, white tags, or no tags. Surveyors were unable to read the tag numbers during the snorkel survey. At the same time, crews used mobile receivers on the shore to determine how many radio-tagged fish were currently present in the segment of the river being surveyed. Hence, these shore surveys gave an exact count of the number of radio-tagged fish that are located in the segment, some of which were seen (but not identified) by the snorkel team.

About one-third of the fish were double tagged with the white Floy tags for a long-term study on tag loss. Because the snorkel crews are unable to read the tag numbers on the fish, it is impossible to estimate tag loss from the snorkel survey. However, because the interval between the tagging and snorkel surveys is short and the water conditions not too extreme (e.g., no larger flows), tag loss is expected to be small. If tag loss were a concern, it could be estimated using a third color of tags (reserved for double-tagged fish) with the snorkel teams instructed to also note how tags of this third color are on the fish, assuming that they now do not overlook tags.

Because the data are sparse when considering all 12 segments, it was reduced to 6 segments by combining adjacent segments (Table 2).

### Statistical model

The population is assumed to be closed between the two sampling events, but movement among segments is permitted for all fish. We assume that all fish (regardless of tag type) have the same probability of movement among the segments and of being sighted by the snorkel surveyors. Furthermore, we assume that that there is 100% detectability of radio-tagged fish by the shore crew within the segment during the second survey. Because not all segments were surveyed simultaneously during the snorkel surveys, it is possible for some radio-tagged fish that were missed because they have changed segments between snorkel surveys. Presumably, the same pattern of “leakage” occurs for all fish – an adjustment to the estimated population size to account for this leakage will need to be carried out.

Using the notation in Table 1, the number of radio-tagged fish that are observed to move between segment *i* and segment *j* between the two surveys is modeled as a multinomial distribution with a separate distribution for each row:

**Table 1:** Notation used in the paper.

Statistics	
*S*	The number of segments on the river
*R*_*ij*_	The number of radio-tagged fish released in segment *i* and detected by the shore crew in segment *j*. We let segment *S* + 1 represent radio-tagged fish not detected by the shore crew (leakage). Because not all segments are surveyed at the same time, it is possible for some of the radio-tagged fish to be “missed” by the shore crew, for example, a fish moves from segment 1 to segment 2 when segment 1 is being surveyed and then back to segment 1 when segment 2 was surveyed. We use the *S* + 1 segment for the second sample to represent fish that missed during the second sample because of such movement. *i* = 1, …, *S*; *j* = 1, …, *S* + 1
*R*_*i*_	The number of radio tags applied in segment *i* during the first sample. 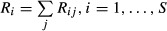
*AR*_*j*_	The number of radio-tagged fish available in segment *j* that could be seen by the snorkel team. Because we assume 100% detectability of radio-tagged fish by the shore crew, the number of radio-tagged fish available to be seen by the snorkel team in segment *j* is known to be 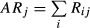
*u*_*j*_	The number of fish seen by the snorkel team in segment *j* without any tags
*WT*_*i*_	The number of white tags applied in segment *i*
*wt*_*j*_	The number of white tags seen in segment *j* by the snorkel teams
Parameters	
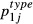	The probability that a fish currently in segment *j* is captured by angling and has a *type*=*radio* or *type*=*white* (Floy) tag applied. This parameter is used only in the simulation study
*p*_*2j*_	The probability that a fish currently in segment *j* is seen by the snorkel team. This is assumed to be the same for green- (radio), white-, and untagged fish
*θ*_*ij*_	The probability of the movement between segment *i* and segment *j* between the two surveys. While it is possible that radio-tagged fish could be detected from shore more than once in two different segments, this did not happen in this experiment and so also assume that 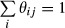 for each row. The *S* + 1 column represent movement of fish that is “missed” as outlined earlier. *i* = 1,…,*S*; *j* = 1,…,*S* + 1
*U*_*j*_	The number of untagged fish in the population in segment *j* during the snorkel survey
*U*	Total number of untagged fish in the population over all segments during the snorkel surveys. 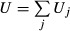 . *U* may be less than the total number of untagged fish because some untagged fish can move between segments among the snorkel surveys and not be subject to catch. An adjustment will make to account for this leakage to estimate the total unmarked population size at the time of initial marking


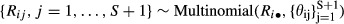


Modeling movement of the white-tagged fish is more difficult because no information is available on the individual fish. We adopt a marginal likelihood approach where we model the (hidden) number white tags that move between segment *i* and segment *j* as a Poisson random variable


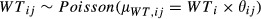


The available number of white tags in segment *j* is the sum of these latent movements:


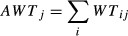


Finally, the observed number of white tags seen by the snorkel team is again modeled as a Poisson random variable


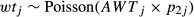


Given the small number of recaptures, the error in estimation introduced by these approximations is expected to be small.

Because of the sparse matrix of movements (Table 2), we place constraints on the movement matrix by assuming that a fish in segment *i*, at the time of marking, can only move a maximum of 2 segments upstream or downstream from the initial segment and that all fish regardless of where released have the same restriction. We do not allow fish to “escape” by moving before or after the last segment and restrict the movement rates accordingly.

**Table 2:** Summary of the data 1 from the Upper Fording River, 2012.

Segment of release	White tags applied	Green (radio) tags applied	Segment at second sample						

			1–2	3–4	5–6	7–8	9–10	HP	Not seen

			Green (radio) tags detected						
1–2	21	8	5	1	0	0	0	0	2
3–4	36	10	0	5	4	0	0	0	1
5–6	27	11	0	0	9	0	0	0	2
7–8	37	18	0	1	1	14	1	1	0
9–10	14	9	0	0	0	0	3	5	1
HP	16	4	0	0	0	0	0	4	0
Green (radio) tags available for snorkel team			5	7	14	14	4	10	
Green (radio) tags seen by snorkel team			4	7	11	10	2	1	
White tags seen by snorkel team			6	7	10	14	4	13	
Untagged seen by snorkel team			375	105	194	51	106	165	

1 One green (radio)-tagged fish found dead soon after release has been removed.

**Table 3:** Comparison of estimates computed in various ways.

Estimate	Radio tags only	White tags only	Radio and White tags combined
Pooled-Petersen	1,809 (*SE* 185)	3,002 (*SE* 310)	2,546 (*SE* 194)
Stratified Petersen^1^	2,026 (*SE* 317)	3,073 (*SE* 420)	2,620 (*SE* 280)
Bayesian hierarchical^1^	1,901 (*SD* 217)	3,022 (*SD* 366)	2,571 (*SD* 223)
Movement model			2,441 (*SD* 311)

1 Assuming no movement among strata.

We place a Dirichlet prior with values of (2,3,5,3,2) for the movement probabilities based on biological knowledge of the species behavior. This gives a one-third prior probability of staying in the same segment after release (the middle entry); a 20% prior probability of moving one segment higher or lower on the river (the 2nd and 4th entries); and the remaining prior probability of moving 2 segments lower or higher in the river.

The number of green (radio)-tagged fish seen by the snorkel team is assumed to be binomially distributed


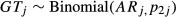


Similarly, the number of untagged fish seen by the snorkel survey is assumed to follow a binomial distribution


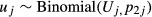


The same survey methods were used on all segments of the river with approximately the same effort in each segment. We expect that the tagging and recapture probabilities were comparable across segments, and because the number of fish is relatively small (especially for the radio-tagged fish), we model the logit of the capture probabilities as exchangeable normal random variables with common mean and variance, that is,


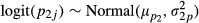


The prior for the mean (on the logit) scale is a logistic distribution with a mean of 0 and a scale parameter of 1, which makes the prior on the probability scale approximately flat. The prior for the variance (on the logit scale) follows an *InverseGamma* (.001,.001) distribution.

A discrete uniform prior, U(1,2000), was used for the number of unmarked fish in each segment. The upper bound was chosen to be about 3–4x larger than expected, based on the densities of fish seen in other streams. The estimate of the total unmarked population size is found as *U* = ∑ *U*_*j*_. Because not all segments are searched simultaneously, it is possible for fish to move among segments between the snorkel surveys on different segments. For example, only 54 of the 59 radio tags (Table 2) were detected on the segments during the snorkel surveys indicating that 5 radio-tagged fish were missed. Consequently, an (admittedly ad hoc) adjustment to the total number of unmarked fish is made to account for this “leakage”


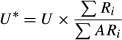


Finally, the total population size is found by adding the total number of radio and white tags applied.

All computations were performed using R (R Core Team. 2012) and OpenBugs (Lunn et al. 2009), and the source code is available on the journal Web site.

## Results

A simple pooled-Petersen estimate (Table 2) is formed by ignoring stratification and pooling over both tag types, and the estimated population size is 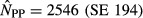 fish. However, a contingency table comparing the marked fraction in each stratum shows strong evidence that the marked fraction is different across the strata (*p* = .002) and the pooled-Petersen estimator may be biased. A simple stratified-Petersen estimate (Table 2) is formed by assuming no movement among strata (which of course is untenable) and by adding the separate estimate from each stratum. This gives a similar estimate of 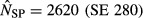 fish. We compute the estimates of the population size using only the radio-tagged fish, only the white-tagged fish (assuming no movement among strata), and the combined radio- and white-tagged fish (again assuming no movement among the strata). All three types of estimators were comparable when the same types of tags were used.

The estimate of population size from our Bayesian movement model is 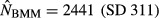, which is virtually the same as the estimates above.

Estimates of catchability among the segments during the snorkel survey range from 0.47 to 0.55 compared to an empirical estimate of average catchability of 0.48 based on the total recaptures of tags compared to those released.

The estimated movement probabilities (in terms of segments from the segment released) are as follows: 0.05 (SE .02) for 2 (pooled) segments downstream; 0.06 (SD .03) for 1 (pooled) segment downstream; 0.58 (SD .06) for remaining in the (pooled) segment of release; 0.18 (SE .04) for 1 pooled segment upstream; and 0.11 (SD .03) for 2 pooled segments upstream. There is a tendency for fish to move slightly further upstream between the two surveys.

### Simulation study

**Figure 1:**
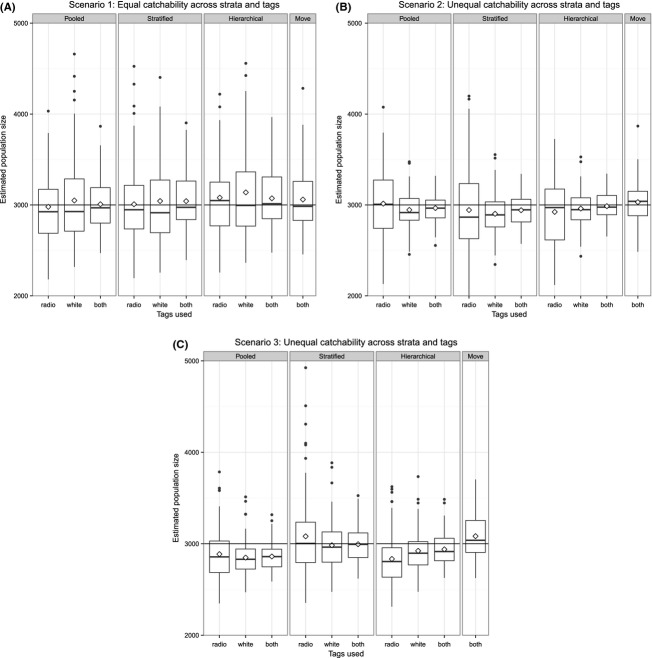
Comparison of the estimators from the simulation study under three scenarios (A, B, C as outlined in text). Actual population size was 3000 fish. Diamonds represent the mean of the estimates over 100 simulations.

A small simulation study was performed to assess the performance of the proposed method and the nine other estimators used in this paper. In each of the scenarios below, 100 simulated data sets were created. For each simulated data set, the estimates (either the MLE or the mean of the posterior) and a measure of precision (either the SE or SD) were obtained. Box plots of the estimates under the scenarios are shown in Fig. 1. A comparison of the actual variation in the estimates across the simulations and the average measure of precision showed that the latter were good estimates of the former and are not shown.

In all scenarios, a population of 3000 fish was distributed across 6 segments using a multinomial distribution with equal probability for each segment. Fish moved among segments between surveys using the movement probabilities estimated in the previous example (e.g., there is 0.58 probability that a fish will remain in the same segment).

In Scenario 1 (Fig. 1a), fish were tagged with either a radio tag or a white tag according to a Bernoulli process with the initial capture (and tagging) probability for both tag types of 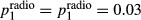 in all strata (no fish was tagged with both types of tags). The snorkel survey had a *p*_2_ = 0.40 probability of detecting a fish regardless of strata, if tagged, or tag type. This scenario satisfies one of the several possible conditions where a pooled-Petersen estimator will be unbiased, that is, equal catchability for all fish in either sampling event. It is not surprising that Figure 1a shows that all estimation methods performed comparably. The uncertainty in the estimator is smaller when both the white and radio tags are used (as they behave identically in this scenario), and there is no reason to use any estimator except the pooled-Petersen with both types of tags.

In Scenario 2 (Fig. 1b), capture efficiency varied across strata, tag types, and sampling occasions, but the capture probabilities during the snorkel survey were independent of those in the tagging survey. Fish were tagged with a radio tag with an average probability of 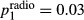 but this varied across strata with a standard deviation of 0.50 on the logit scale (giving a range of 0.01 to 0.08 across the strata). Fish were tagged with a white tag with an average probability of 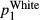 = 0.09, but this varied (independently of the radio-tagging probability) also with a standard deviation of 0.50 on the logit scale (giving a range of 0.03 to 0.21 across the strata). Finally, fish were seen in the snorkel survey with an average probability of *p*_2_ = 0.40, but this varied also with a standard deviation of 0.50 on the logit scale (giving a range of 0.20 to 0.60 across the strata). Even though the capture probabilities vary between sampling events and across strata, for most fish, the two probabilities were independent and so the bias from pooling is small relative to the uncertainty in the estimates. Again, all methods are comparable.

Finally, in Scenario 3 (Fig. 1c), strata 1, 3, and 5 had 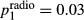, 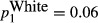, and *p*_*2*_ = 0.3, while strata 2, 4, and 6 had 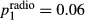, 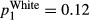, and *p*_2_ = 0.60. This corresponds to some segments where conditions are easier or harder to catch fish, and now the heterogeneity in catchability is no longer independent among fish. Under these conditions, the pooled methods will have a negative bias in the estimates of population size. The stratified methods have less bias only because the majority of fish stayed within the segments of tagging and so fewer fish exhibited individual heterogeneity. The hierarchical methods pulled the strata capture rates towards the mean and so also exhibited some bias, especially with the small sample sizes for radio tags where the shrinkage to the mean would be largest. The methods of this paper performed reasonably well.

We also tried a scenario (not shown) where tagging with white tags was concentrated into strata 1, 3, and 6. Unlike the case where white tags are distributed evenly across the strata, this unequal distribution makes it much easier to detect and estimate movement, that is, white-tagged fish seen in the snorkel survey in strata where no white tags were applied must have moved there from adjacent strata. Stratified and hierarchical estimators often fail because now it is possible to detect more tagged fish in the snorkel survey than fish released in that stratum. The movement model also outperformed the pooled estimators similarly to seen in Scenario 3.

## Discussion

For this particular application, the main focus is on the population estimation rather than estimating movement. Movement parameters are nuisance parameters that need to be modeled because of differences in catchability among the strata. Perhaps uniquely to this study, the movement patterns are also needed to model the availability of the batch marks for the snorkel team as not all segments can be surveyed simultaneously. Based on the radio tags, about 10% of tagged fish are unavailable to the snorkel teams. Because this unavailability acts similar to “mortality” (fish leave the population between tagging and recapture), simple Petersen estimates are still unbiased for the population size at the time of the first survey. The movement model, however, requires an ad hoc adjustment.

The hierarchical model improves estimation through “partial pooling.” Because the number of radio tags available to the snorkel teams in each segment is small, estimates of the recapture rate in each segment will be unreliable if each segment is treated individually.

**Figure 2:**
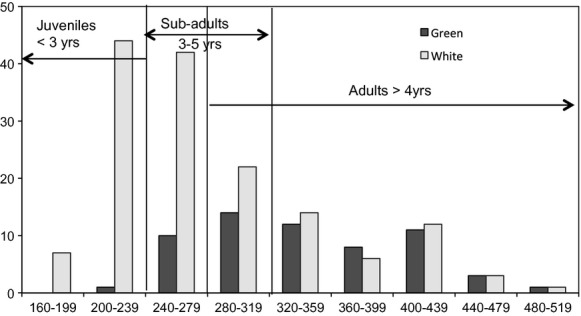
Length frequency of white Floy-tagged fish (*n* = 151) and radio- and green Floy-tagged fish (*n* = 60) illustrating the differences in the representation of juvenile and subadult life-history stages.

There are two (related) unresolved problems in the application of this model to the current study. First, it is evident that the gross resighting rate differs between the radio-tagged (green tags at 59%) and white-tagged fish (at 40%). For this study, it is almost certainly due to differences in the length distribution of tagged fish as shown in Fig. 2. It is evident that radio tags are mostly applied to larger fish compared to white tags and this size may be related to sightability in the snorkel survey. Unfortunately, without information on the exact fish seen by the snorkel team, it is difficult to model catchability as a function of the length of the fish.

Estimates based solely on white-tagged fish are consistently larger than the other estimates. This is not surprising because of both the unavailability of some of the tagged fish to the snorkel team and because, in general, these tags have a lower sighting rate (presumably because they are smaller).

The estimates using the radio-tagged fish only may have biases in the opposite direction for similar reasons.

Does the batch tagging improve estimation? There does not seem to be much of a difference between the stratified and unstratified estimates because the differences in sightability across the strata are fairly small and so there is no advantage of stratification similar to Scenario 1. In other studies where difference in catchability is larger and fixed among strata (similar to Scenario 3), our proposed method will be more useful. The batch marks are still useful as part of the long-term study where these fish can be recaptured in future years and the Floy-tags read.

We also did not use auxiliary information from fixed-station receivers that were also present that show if a radio-tagged fish has passed the station. This could provide information on the location of the “unavailable” fish and improve the modeling, but it is at a coarse level, even after we reduced the number of segment by pooling adjacent smaller segments.

This issue of unavailability will be more of a concern with temporal stratification such as those used in enumeration of smolts using rotary screw traps (Bonner and Schwarz 2011). In some cases, hatchery fish are all adipose-fin clipped (batch-marked) and released en masse. We expect batch marks and the methods in this paper to be much more effective in these cases as detection of batch marks then provides good information on the movement rates.
